# Coronavirus disease COVID-19 pandemic and the Declaration of Public Health Emergency in Brazil: administrative and epidemiological aspects

**DOI:** 10.1590/0037-8682-0227-2022

**Published:** 2022-05-20

**Authors:** Wanderson Kleber de Oliveira, Luciano Pamplona de Góes Cavalcanti, Julio Croda

**Affiliations:** 1 Supremo Tribunal Federal , Secretaria de Serviços Integrados de Saúde, Brasília, DF, Brasil.; 2 Ministério da Defesa , Hospital das Forças Armadas, Departamento de Ensino e Pesquisa, Brasília, DF, Brasil.; 3 Centro Universitário do Planalto Central Apparecido dos Santos, Faculdade de Medicina, Brasília, DF, Brasil.; 4 Universidade Federal do Ceará, Programa de Pós-Graduação em Saúde Coletiva, Fortaleza, CE, Brasil.; 5 Centro Universitário Christus, Faculdade de Medicina, Fortaleza, CE, Brasil.; 6 Universidade Federal do Ceará, Programa de Pós-graduação em Patologia, Fortaleza, CE, Brasil.; 7 Universidade Federal de Mato Grosso do Sul, Faculdade de Medicina, Campo Grande, MS, Brasil.; 8 Yale School of Public Health, Department of Epidemiology of Microbial Diseases, New Haven, USA.; 9 Fundação Oswaldo Cruz, Campo Grande, MS, Brasil.

October 2001, after the September 11 attack, mails contaminated by *Bacillus anthracis* were sent to American agencies worldwide, including those in Brazil^1^. Anthrax was included in the compulsory notification list, and laboratories were required to notify of the existence of agent samples^2^. Global public health enters a new era of preparedness and response to public health emergencies. The distrust caused by terrorist acts escalated during the severe acute respiratory syndrome SARS epidemic, caused by a coronavirus, which emerged in China in late 2002. Available information at the time was insufficient, resulting in lack of preparation worldwide. Therefore, the revision of the International Health Regulations IHR, which had started in 1995, was completed in 2005.

The IHR 2005 introduced the concept of Public Health Emergency of International Concern PHEIC, which was applied in the declaration of the World Health Organization WHO on January 30, 2020, with regard to the coronavirus disease 2019 COVID-19. A PHEIC can be declared in a time of any extraordinary event that poses a risk to the public health in other countries due to the international spread of a disease, injury, or occurrence with the potential to cause disease e.g., disasters or terrorist attacks, and that require a coordinated international response^3^. 

Since the implementation of IHR, six international emergencies have been declared by the WHO: influenza A H1N1, lasted 472 days 04/25/2009-10/08/2010; wild poliovirus, active for more than 7 years 05/05/2014-current; Ebola, lasted 599 days 08/08/2014-03/29/2016; Zika virus, lasted 291 days 01/02-18/11/2016; Ebola, 375 days 06/17/2019-06/26/2020; and COVID-19, ongoing for more than 2 years 01/30/2020-current^4^. 

Although Brazil was the third country to sign the IHR in 2005, only in 2020 was the translated and approved text by the National Congress promulgated and inserted into the national legal framework, through Decree No. 10,212 on January 30, 2020, expanding the legal value of measures related to health situations 3. However, the concept of the Public Health Emergency of National Importance PHENI arose on November 17, 2011, in Decree No. 7,616 that expatiated on the PHENI declaration by three different situations: epidemiological, disasters, or lack of assistance to the population^5^. To enable the structuring of the States, Federal District, and Municipalities, the concept is included in the National Health Surveillance Policy NHSP, through Resolution No. 588 of July 12, 2018, which broadly defines a Public Health Emergency PHE as a situation that demands urgent measures to prevent, control, and contain risks, damages, and harm to public health^5^ ^,^ ^6^. 

The characterization of the geographical and administrative amplitude of PHE as being of national, regional, state, municipal, or local importance is conditioned to the epidemiological dimension of the public health event, defined in Consolidation Ordinance No. 4, as a situation that may constitute a potential threat to public health, such as the occurrence of an outbreak or epidemic, disease or injury of unknown cause, and alteration in the epidemiological clinical pattern of already known diseases. Thus, it becomes necessary to evaluate and characterize whether the situation would constitute a public health emergency through the evaluation of the dissemination potential, magnitude, seriousness, severity, transcendence, and vulnerability, as well as epizootic diseases or injuries resulting from disasters or accidents^7^. Therefore, epidemiological criteria allow the declaration of a PHENI, even without case confirmations for a certain disease within the Brazilian territory-i.e., this tool can be evoked to prepare the system against a potential threat to national public health, as occurred in Ordinance No. 188, of February 3, 2020, aiming to coordinate a response against the threat of COVID-19. At the time, no confirmed cases had been reported in Brazil, and the consequences were uncertain^8^. 

In public health, emergencies may be characterized as epidemics but may be confined to a region and it would not be declared as such. Thus, the administrative act of declaring an emergency should be dissociated from the epidemiological evaluation classification in an isolated case, outbreak, epidemic, pandemic, or endemic. If the event does not constitute a threat, or the criteria are no longer met after the PHENI has been declared, the instrument must be revoked to preserve it for future situations. This instrument acts as a “broad spectrum remedy” to enable action streamlining, mobilizing the attention of managers, and attracting financial, human, and material resources. Thus, it should last long enough for epidemiological conditions to become favorable and/or the main needs to be met. 

Epidemiological situations can only be declared by the Minister of Health, on the recommendation of the Health Surveillance Secretariat. In the event of disasters, it may be declared at the request of the Ministry of Regional Development, and in non-assistance cases, by means of a request from the Executive Branch of the affected federated unit^5^. 

At the time of COVID-19’s PHENI declaration, the situation met four of the criteria: risk of national dissemination, produced by an unexpected infectious agent, high severity, and extrapolation of the responsiveness of the States and the Federated District^8^. Two years later, the current situation is essential to contextualize some of the aspects so that the change in administrative status is configured rationally, rather than passionately. 

 Based on the epidemiological context, knowledge of SARS-CoV-2 and its variants, the level of evidence, and the current Unified Health System **Sistema Único de Saúde - SUS** structure as premises for assessing the COVID-19 pandemic, and maintaining the PHENI declaration and projection until August 2022 Figure 1, the following should be considered:

**FIGURE 1:**
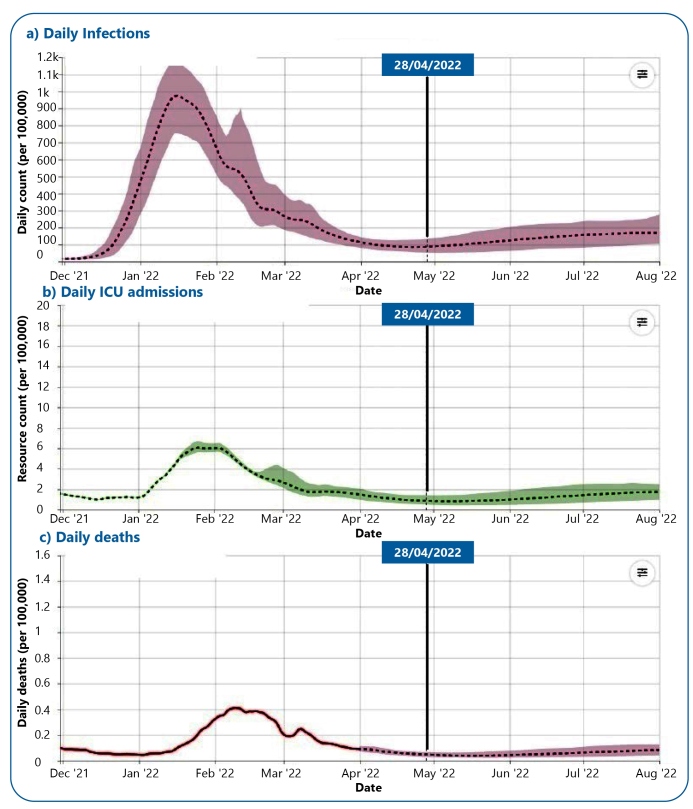
Daily number of infections **a**, ICU admissions **b**, and deaths **c** per 100,000 inhabitants, recorded for COVID-19 until March 31, 2022, and estimated until August 1, 2022. **Source:** IHME-https://covid19.healthdata.org/-28/04/2022

1. **Risk of national dissemination:** According to the Oswaldo Cruz Foundation Fiocruz, at the beginning of April 2022, the Omicron variant and its subvariants are predominant throughout the Brazilian territory. Therefore, this condition does not apply in the current context, unless the threat of arising new variants is considered. Nonetheless, it must be pragmatic and focused on facts and concrete evidence.
2. **Produced by unexpected infectious agents:** SARS-CoV-2 and its known and described variants are regularly monitored by the Fiocruz Genomic Network, Rede Corona-ômica Brazilian Ministry of Science and Technology, Rede Alerta das Variantes Butantan Institute, and Private institutions DB Molecular, HLAgyn and DASA laboratories. Several instruments have already been developed, such as a Surveillance Guide, clinical protocols, and case definitions; the disease is listed in the compulsory notification list. Therefore, this condition no longer seems to fit in the current context.
3. **Reintroduction of an eradicated disease:** This has not been applied since the beginning of the pandemic. However, given the low vaccination coverage against polio, it can be evoked on any threat of reintroduction, as seen recently in Israel^9^. However, for COVID-19, this is not a valid criterion.
4. **High severity:** Brazil is one of the countries most affected by the COVID-19 pandemic. At the end of epidemiological week EW 15 through 16 April 2022, over 30 million cases and over 662,000 deaths were reported. However, in recent weeks the mortality rate, which has already exceeded 10 deaths per 100,000 inhabitants, has fallen to approximately 0.4 deaths per 100,000 inhabitants - the lowest rate since EW15/2020. The vaccination coverage 80% of the eligible population aged 5 years or more, with 38% having received one or two booster shots, 80% of which among the elderly significantly reduces the severity and impact of the disease in Brazil. Moreover, the clinical capacity to identify, manage and support life became more robust after the successive case waves. Therefore, to maintain the PHENI declaration, we can also consider this component questionable.
5. **Extrapolate the capacity of the state management of the SUS:** Surveillance and emergency response capacities have improved, considering: the expansion of Center for Strategic Health Surveillance Information CSHSI units throughout Brazil; the expansion of the capacities of the Central Public Health Laboratories Laboratórios Centrais de Saúde de Pública - Lacens, Fiocruz, Butantan Institute, universities and private initiatives with sequencing structures in the majority of them; the greater availability of RT-PCR and antigen tests; the training of professionals and surveillance through the basic, intermediate, and advanced levels of Field Epidemiology Training Program FETP in Brazil EpiSUS; training of primary and specialized health care professionals; greater availability and lower cost of personal protection equipment; greater expansion of specialized and ICU beds in the recent history of SUS; and extra budgetary resources to reduce the impacts of the pandemic^10^ ^,^ ^11^ ^,^ ^12^ ^,^ ^13^.

Given these aspects, the extrapolation of capacity within the context of preparation for future short-term scenarios should be evaluated: 

**Scenario 1:** Reduction in the number of cases and deaths until the agent is no longer characterized as a public health issue, like common coronaviruses.

**Scenario 2:** Maintenance of cases and deaths that characterize COVID-19 as a public health problem, implying the maintenance of regular surveillance and response strategies, like other endemic diseases and within structural capacity.

**Scenario 3:** Occurrence of new variants that alter the responsiveness, requiring new PHENI declarations to coordinate responses within the SUS. 

Whatever the scenario, the administrative public health emergency of national importance and epidemiological epidemic, pandemic, and endemic dimensions should be understood as complementary, but not directly conditioned. An emergency can be declared even without a confirmed case, and an epidemic may occur without this declaration. All will depend on the risk assessment probability and impact from the threats external factors and vulnerabilities internal factors to the SUS in Brazil. This scenario analysis is not restricted to Brazil and the WHO in the near future should discuss and reflect on the risk assessment to declare the end of the public health emergency.
